# Transgelin-2 is a novel target of KRAS-ERK signaling involved in the development of pancreatic cancer

**DOI:** 10.1186/s13046-018-0818-z

**Published:** 2018-07-24

**Authors:** Yan Sun, Wenfang Peng, Weiwei He, Man Luo, Guilin Chang, Jiping Shen, Xiaoping Zhao, Yu Hu

**Affiliations:** 1 Department of Geriatrics, Zhongshan Hospital, Fudan University, Shanghai, 200032 China; 2 Department of Endocrinology, Shanghai Tongren Hospital, School of Medicine, Shanghai Jiao Tong University, Shanghai, 200336 China; 3 Department of Thoracic Surgery, Sixth People’s Hospital, School of Medicine, Shanghai Jiao Tong University, Shanghai, 200233 China; 4 Department of Nuclear Medicine, Ren Ji Hospital, School of Medicine, Shanghai Jiao Tong University, Shanghai, 200025 China

**Keywords:** PDAC, Transgelin-2, KRAS, ERK

## Abstract

**Background:**

The KRAS mutation is the driving force of pancreatic ductal adenocarcinoma (PDAC). Downstream effectors of KRAS signal pathways are crucial to the development of PDAC. The purpose of this study was to investigate the relationship between KRAS mutation and transgelin-2. Transgelin-2 is highly expressed in PDAC tissues compared with adjacent normal tissues. The underlying mechanism for upregulating transgelin-2 is largely unknown.

**Methods:**

Expression of transgelin-2 was analyzed by microarray data and qRT-PCR. The effect of KRAS signaling on transgelin-2 expression was examined in PDAC cells in the presence or absence of the ERK inhibitor. The interaction of transgelin-2 with ERK was confirmed by immunoprecipitation. ERK-mediated Phosphorylation of transglein-2 was detected by in vivo and in vitro kinase assays. The gain-of-function and loss-of-function approaches were used to examine the role of phosphorylation of transgelin-2 on cell proliferation. Phosphorylation of transgelin-2 was detected by immunohistochemistry in PDAC tissues.

**Results:**

Here we found transgelin-2 expression was induced by KRAS mutation. In the case of KRAS mutation, ERK2 interacted with 29–31 amino acids of transgelin-2 and subsequently phosphorylated the S145 residue of transgelin-2. S145 phosphorylation of transgelin-2 played important roles in cell proliferation and tumorigenesis of PDAC. In addition, S145 phosphorylation of transgelin-2 was associated with a poor prognosis in patients with PDAC.

**Conclusions:**

This study indicated that KRAS-ERK-mediated transeglin-2 phosphorylation played an important role in the development of PDAC. Inhibition of transgelin-2 phosphorylation may be a potential therapeutic strategy for targeting PDAC with KRAS mutation.

## Background

Pancreatic cancer is the third leading cause of cancer-related death in the United States [1]. Pancreatic cancer is a rapidly fatal disease with a 5-year survival rate of less than 5%. Pancreatic ductal adenocarcinoma (PDAC) accounts for 95% of pancreatic cancer [2]. There is still no effective treatment for advanced PDAC [3]. A better understanding of the role of driver mutations in the development of PDAC is likely to find new therapeutic targets.

More than 90% of PDACs carry mutated *KRAS* alleles [4]. KRAS mutations have been shown to play a key role in the development of PDAC [5]. The most common mutation is the constitutively active KRAS^G12D^ allele. KRAS^G12D^ mutation is essential for the initiation and maintenance of pancreatic cancer [6]. Although KRAS mutations have been identified as a driver of PDAC, KRAS targeted therapy has not been successfully developed. Direct inhibition of KRAS has proven clinically challenging. Inhibition of KRAS downstream targets is an effective strategy for targeting KRAS mutations. KRAS activates different downstream effectors in a context specific manner. The KRAS-driven signal network is different between PDAC, non-small cell lung cancer (NSCLC) and colon cancer [7]. Therefore, it is necessary to clarify the precise molecular mechanism of KRAS in the development of pancreatic cancer.

Transgelin-2 belongs to the family of actin binding proteins (ABPs) and has been characterized as a smooth muscle cytoskeletal protein. In recent years, dysregulated expression of transgelin-2 has been reported in different types of cancers. Up-regulation of transgelin-2 was observed in pancreatic cancer [8], colorectal cancer [9], lung adenocarcinoma [10, 11] and cervical squamous cell carcinoma [12]. Previously, we found that transgelin-2 is highly expressed in PDAC tissues compared with adjacent normal tissues. High level of transgelin-2 is associated with poor prognosis in patients with PDAC [8]. In contrast, down-regulation of transgelin-2 was observed in the tissues of Barrett’s adenocarcinoma patients [13]. Therefore, specific upstream factors are involved in regulating the context-dependent expression of transgelin-2. Driver gene mutations play a key role in tumorigenesis. In general, cancer contain 2–8 of these key mutations [14]. Although transgelin-2 is known to be involved in the development of cancer [15], the relationship between transgelin-2 and driver gene mutation is not fully understood.

In the present study, we analyzed the relationship between KRAS and transgelin-2 in PDAC. We found that the protein stability of transgelin-2 was regulated by KRAS. ERK-mediated phosphorylation resulted in accumulation of transgelin-2 protein. These findings indicate transgelin-2 is a downstream target of KRAS signaling. KRAS-ERK-transgelin-2 axis may be explored for targeted therapy of PDAC.

## Methods

### Patients

This work was done with the approval of the Ethics Committee of Zhongshan Hospital. A total of 114 patients diagnoses with pancreatic cancer between 2003 and 2009 were enrolled in the study. Clinical characteristics including age, gender, anatomical location of tumor, histology of the tumor, lymph node involvement and metastasis status, were obtained from patient records. Patients who did not reach the outcome under study were censored at the date of their last visit. For the analyses of overall survival, each patient’s time began on the date of diagnosis and ended on the date of death or on the date last seen alive.

### Immunohistochemical staining

Immunohistochemical staining of paraffin sections for transgelin-2 or SREBP-1 protein was performed with an LSAB kit (DAKO, Marseilles, France), using p-145-transgelin-2 antibody (dilution, 1:500) The sections were incubated in 3,3′ diaminobenzide tetrahydrochloride with 0.05% H_2_O_2_ for 3 min. Immunostaining scores were independently evaluated by three pathologists. Semi-quantitative scores were used to analyze antibody immunostaining. Intensity of staining was categorized into −, +, ++ or +++, denoting negative (0), weak (1), moderate (2) or strong staining (3). Extent of immunostaining was categorized into 0 (< 10%), 1 (10–25%), 2 (26–50%) or 3 (> 50%) on the basis of the percentage of positive cells. Three random microscopic fields per tissue were calculated. The final score of expression level was determined by the formula: final score = intensity score × percentage score. The final score was ranged from 0 to 9. The final score of ≤3 was defined as low expression, and > 3 as high expression.

### KRAS mutation analysis

KRAS mutation was assessed using the Sanger sequencing. Formalin-fixed, paraffin-embedded tissue were taken, and 2 to 3 unstained 10-μm sections were used for DNA extraction. Genomic DNA was extracted using QIAamp DNA FFPE Tissue kit (Qiagen, Hilden, Germany). DNA were amplified in a HotStarTaq Master Mix (Qiagen) using the primers 5’-AAAAGGTACTGGTGGAGTATTTGA-3′ and 5’-CATGAAAATGGTCAGAGAAACC-3′. Cycling conditions of the PCR were as follows: initial denaturation at 95° for 5 min, followed by 35 cycles of 95 °C for 45 s, 58 °C for 45 s, 72 °C for 1 min and a final extension at 72 °C for 5 min. The purified PCR product was used for sequencing.

### Cell lines and transfection

The cell lines BxPC-3 and HPDE6-C7 were purchased from the cell bank of the Chinese Academy of Sciences (Shanghai, China), and the other cells were obtained from the ATCC (Manassas, VA, USA).. The PDAC cell lines were maintained in DMEM or RPMI 1640 medium (Invitrogen) supplemented with 10% FBS (Invitrogen), 100 U/ml penicillin (Invitrogen), and 100 mg/ml streptomycin (Invitrogen). All transfections were performed with Lipofectamine 2000 (Thermo Scientific, Waltham, MA, USA) or Lipofectamine RNAiMAX (Thermo Scientific) transfection reagents. Cells were cultured until 40–50% confluence at the time of transfection. At 24–48 h after transfection, cells were harvested for quantitative PCR or western blot analysis. For gene knockdown experiments, control cells were incubated with OPTI-MEM and transfection reagent (vehicle group), or with OPTI-MEM and transfection reagent plus no-silencing siRNA (siRNA-NC group). The KRAS siRNA sequences are as follows: sense CAGCUAAUUCAGAAUCAUU, antisense AAUGAUUCUGAAUUAGCUG.

### Real-time PCR

Total RNA was isolated and purified using an TRIZOL Reagent (Invitrogen). RNA quality was assessed using NanoDrop 2000 (Thermo Fisher Scientific, USA) and RNA integrity was assessed using electrophoresis through an agarose gel. The first strand cDNA was synthesized using 1 μg of RNA and SuperScript® III Reverse Transcriptase (Invitrogen). qRT-PCR was performed with SYBR Green PCR reagents on an ABI Prism 7900 detection system (Applied Biosystems, CA, USA). RNA levels were normalized to the level of β-actin or GAPDH and calculated as delta-delta threshold cycle (ΔΔCT). The primer sequences were as follows: transgelin-2 forward: GGAGATCTCTCCCCGCA, reverse TCCACTGGATCAGGATCTGC; KRAS forward: -TGACCTGCTGTGTCGAGAAT, reverse TTGTGGACGAATATGATCCAA.

### Cell proliferation

Cell proliferation assay was performed as described previously [16]. Briefly, 10^4^ cells/well were seeded into six-well plates after 24 h transfection. Cell numbers were counted every 24 h. At least three independent experiments were performed. Growth curve assays were performed by counting live cells using trypan blue exclusion.

### Western blotting

Cells were lysed in ice-cold lysis buffer (50 mM Tris-HCl, pH 7.4, containing 150 mM NaCl, 1% Triton X-100, and protease inhibitor cocktail) for 30 min on ice. Lysates were centrifuged at 2,0000 g for 30 min at 4 °C. The supernatant was mixed with SDS loading buffer (100 mM Tris-HCl, pH 6.8, 4% SDS, and 20% glycerol with bromophenol blue) and heated for 5 min. Proteins were separated by 12% SDS-PAGE gel and transferred to PVDF membranes. The membrane was blocked in 5% non-fat milk, and incubated with the intended primary antibody in TBS containing 0.1% Tween 20 (TBS-T) for 3 h. After washing with TBST-T, the membrane was incubated with HRP-conjugated secondary antibody for 1 h. After three washes with TBST-T, bands were visualized by chemiluminescence. The primary antibodies used in this study were: as follow anti-transgelin-2 (Novus Biologicals, CO, USA), anti-β-actin (Cell Signaling Technology, MA, USA), anti-Flag (Sigma-Aldrich, MO, USA), anti-GFP (Abcam, MA, USA), anti-ERK1/2 (Cell Signaling Technology, MA, USA), anti-phospho-ERK1/2-T202/y204 antibody(Cell Signaling Technology, MA, USA), anti-β-tubulin antibody (Abcam, MA, USA), anti-GST antibody (Abcam, MA, USA).

### Co-immunoprecipitation

Collected cells were extracted for 30 min in ice-cold lysis buffer (50 mM Tris-HCl, pH 7.4, containing 150 mM NaCl, 1% Triton X-100) containing protease inhibitor cocktail (Sigma-Aldrich, MO, USA). After centrifuge at 2,0000 g for 30 min at 4 °C, supernantant was pre-cleared by incubation with Protein A/G-Sepharose (Sigma-Aldrich, MO, USA). Pre-cleared supernatants were incubated with transgelin-2 antibody or control IgG for 3 h at 4 °C. Then Protein A/G Sepharose was added to pull down the immune-complex. After washing, the immunoprecipiated proteins were analyzed by SDS-PAGE.

### Fusion protein purification

*E. coli* strain BL21(DE3) was transformed with plasmid. While OD_600_ = 1.6, protein expression was induced by addition of IPTG (1 mM) for 4 h at 30 °C. Bacteria were harvested by centrifugation at 4, 000 g for 10 min at 4 °C. The cell pellets were suspended in GST extraction buffer (20 mM HEPES,pH 7.6,0.5 M NaC,0.5uM EDTA,10% Glycerol,0.5% NP-40) containing protease inhibitors. The supernatant was added with glutathione–conjugated bead slurry to incubate 3 h at 4 °C. After extensive washing with GST wash buffer. The fusion proteins were eluted by glutathione. The purified proteins were analyzed SDS-PAGE with coomassie staining.

### In vitro kinase assay

Hek293T cells were transfected with Flag-ERK2 for 48 h. Then cells were lysed in buffer (50 mM Tris-HCl, pH 7.4, containing 150 mM NaCl, 1% Triton X-100). The cleared supernatant was incubated with ANTI-FLAG M2 Affinity Agarose Gel (Sigma-Aldrich, MO, USA) for 3 h at 4 °C. Then Immune complexes were washed 3 times with lysis buffer and assay buffer (50 mM Tris-HCl pH 7.4, 50 mM NaCl, 10 mM MgCl2, 1 mM MnCl2 and 10% glycerol). The GST-transgelin-2 fusion protein was purified as described previously [17] Kinase reactions were carried out in 50 μl of assay buffer with 50 mM ATP at 30 °C for 1 h. The reactions were stopped by addition of 5X sample buffer, followed by boiling for 2 min The supernatants were analyzed by SDS-PAGE.

### Antibody preparation

The p-S145-transgelin-2 antibody was raised against the synthetic phosphopeptide antigen encompassing ARDDGLFS*GDPNWFP, where S* represent phosphoserine. The peptide was conjugated to keyhole limpet hemocyanin and used to immunize rabbits. Phosphopeptide-reactive rabbit antiserum was first purified by protein A chromatography. The purified antibodies then were passed through a column coupled with the unphosphorylated peptide to deplete antibodies that react with unphosphorylated transgelin-2. The specificity of each antibody was confirmed by ELISA assay.

### Xenograft tumor studies

BALB/c severe combined immunodeficiency mice were purchased from Shanghai Laboratory Animal Center. All experimental procedures using animals were in accordance with the guidelines provided by the Animal Ethics Committee of Zhongshan Hospital. Nude mice were subcutaneously injected with 5 × 10^6^ cells expressing transgelin-2 wild-type and S145A mutant in conjunction with stable knockdown of endogenous transgelin-2 by shRNA on the dorsal flanks, respectively. Tumor growth was assessed with caliper measurement. Tumor volume was calculated according to the following formula: V = (Length x Width^2^)/2.

### Statistical analysis

Normality of variables was tested with Shapiro–Wilk test. Normally distribute continuous variables were expressed as mean ± SD, and categorical variables were summarized as median with interquartile range. Quantitative variables with normal distribution were analyzed with one-way ANOVA with Tukey HSD as a post-hoc test. Comparisons between groups with categorical variables were evaluated by Kruskal-Wallis followed by Dunn test. Correlation analyses between continuous or categorical variables were performed by Pearson’s or Spearman’s, respectively. The association between non-parametric variables was assessed with Chi-square test. Parametric variables were compared with the independent samples t-test. *P* < 0.05 was considered to indicate a statistically significant difference.

## Results

### Transgelin-2 is up-regulated upon KRAS mutation

**Fig. 1:**
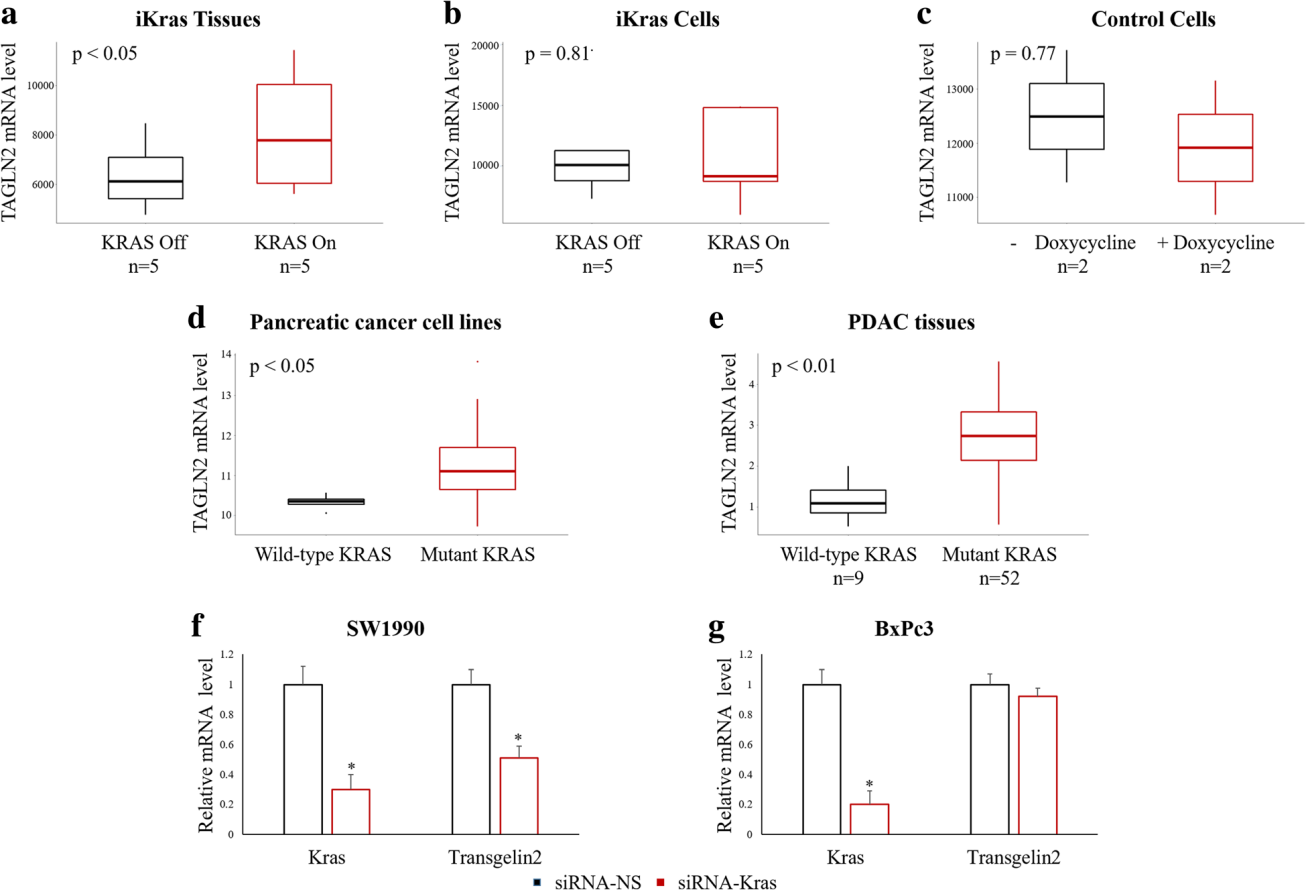
KRAS mutation induces transgelin-2 expression. **a**. The mRNA level of transgelin-2 was analyzed in tissues from tetO_LSL-KRAS^G12D^/p48-Cre/ p53^L/L^ mice. The mice were kept on doxycycline for 2 weeks until obvious tumor formation. Half of the animals were pulled off doxycycline for 24 h. KRAS on indicated induction of expression of KRAS by doxycycline, whereas KRAS off was without KRAS expression. **b**. The mRNA level of transgelin-2 was analyzed in primary cells derived from tetO_LSL-KRAS^G12D^/p48-Cre/p53^L/L^ mouse model. The primary cells were cultured in the presence or absence of doxycycline for 24 h and total cellular RNA was prepared. **c**. The mRNA level of transgelin-2 was analyzed in primary cells derived from tetO_LSL-KRASG12D without crossing with p48cre and p53^L/L^. The primary cells were cultured in the presence or absence of doxycycline for 24 h and total cellular RNA was prepared. **d**. The mRNA level of transgelin-2 was analyzed in CCLE datasets. Cell lines were categorized by KRAS G12 codon status. The “wild-type KRAS” group indicates that the KRAS gene is either wild-type or wild-type at the G12 codon. The “mutant KRAS” group indicates mutations at the G12 codon. Totally, there are 5 cell lines in “wild-type KRAS” group and 30 cell lines in “mutant KRAS” group. **e**. RNA was purified from PDAC tissues. The mRNA level of transgelin-2 was analyzed by qPCR. **f-g**. SW1990 and BxPc3 cells were transfected with siRNA-KRAS or siRNA-NS for 24 h. The mRNA level of transgelin-2 or KRAS were analyzed by qPCR. *p < 0.05 compared with the siRNA-NS group

In previous reports, we have shown increased expression of transgelin-2 in pancreatic cancer tissues compared with adjacent normal tissues [8]. Since KRAS mutations are the most detrimental of all genetic abnormalities in PDAC [5], we asked whether transgelin-2 expression correlated with *KRAS* gene status. In an elegant study, Haoqiang Ying, et al. establish a mouse model in which KRAS^G12D^ expression is under the control of the tet operon and the LSL cassette (tetO_LSL-KRAS^G12D^) [18]. By analyzing Haoqiang Ying ‘s dataset, we found that the level of transgelin-2 in doxycycline-induced mice was significantly higher than that in non-doxycycline-induced mice, suggesting that the KRAS^G12D^ mutation promotes the expression of transgelin-2 (Fig. 1a). Induction of KRAS^G12D^ expression in primary tumor cells from the tetO_LSL-KRAS^G12D^ model resulted in a slight increase in transgelin-2 levels but did not reach statistical difference (Fig. 1b). In primary tumor cells from mice of tetO_LSL-KRAS^G12D^ that did not cross with p48^cre^, doxycycline treatment had no effect or even slightly decreased transgelin-2 (Fig. 1c). Next, we analyzed the dataset of the Cancer Cell Family Encyclopedia (CCLE) [19] to further investigate the relationship between expression of transgelin-2 and KRAS mutation in PDAC cells. We found that the level of transgelin-2 was significantly higher in KRAS G12 mutant pancreatic cancer cell lines than in wild-type or other types of KRAS (G13D, Q61R, I171M, A146T, etc.) (Fig. 1d). In order to verify the relationship between expression of transgelin-2 and KRAS mutation, tissues from 61 patients with PDAC were analyzed. The overall percentages of wild-type and 12th codon mutation of KRAS were 14.8 and 85.2%, respectively, consistent with previous report [7]. Our analysis found that the mRNA level of transgelin-2 in KRAS mutant PDAC tissues were significantly higher than those in wild-type KRAS tissues (Fig. 1e). The correlation between KRAS mutation and transgelin-2 expression was further confirmed in KRAS knockdown cells. In KRAS mutant PDAC cells (Fig. 1f), but not KRAS wild-type cells (Fig. 1g), we found that KRAS knockdown reduced the mRNA level of transgelin-2. These results indicate that KRAS mutation induce the expression of transgelin-2.

### KRAS induces the accumulation of transgelin-2 protein via ERK

**Fig. 2:**
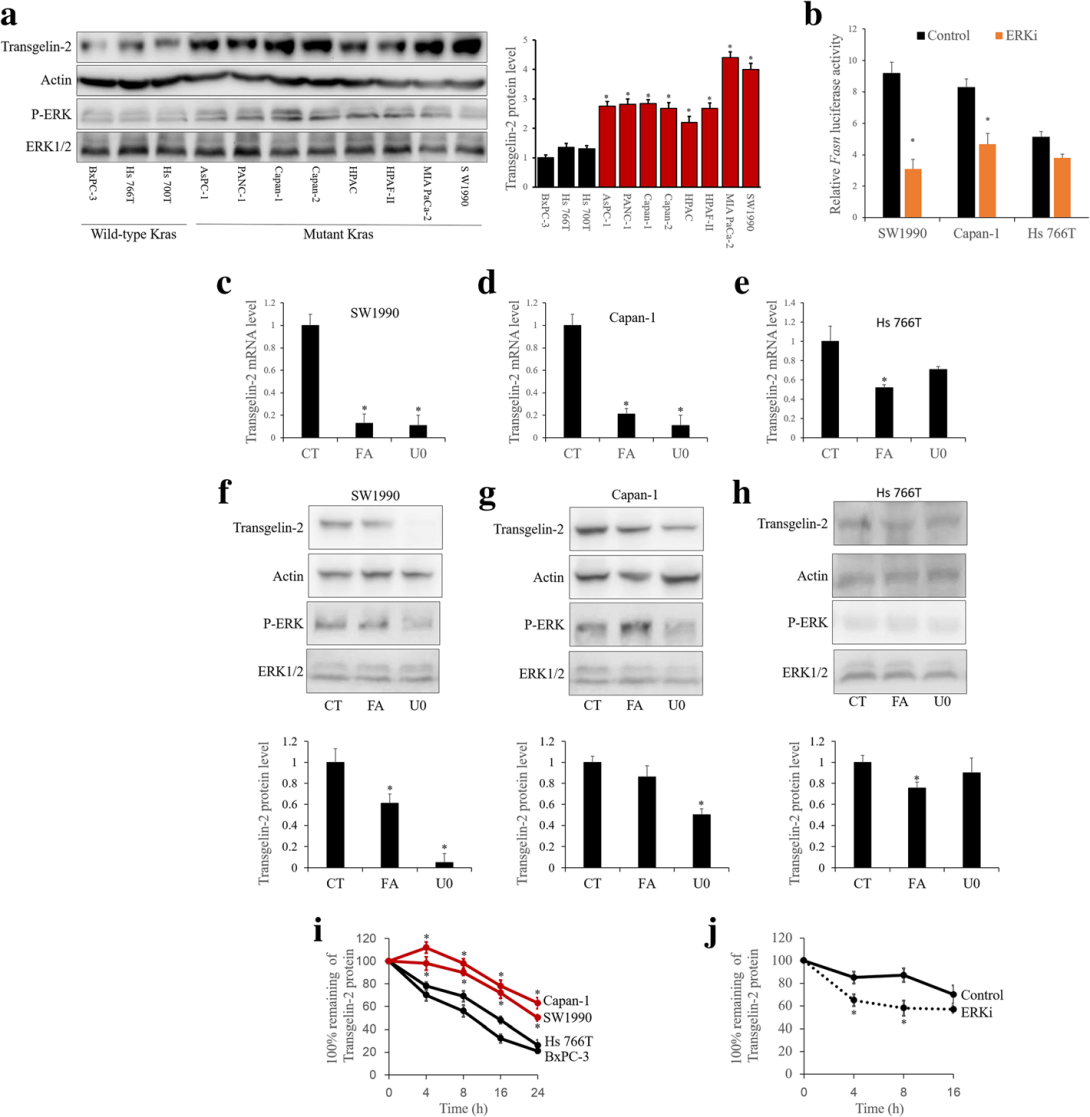
KRAS mutation promotes accumulation of transgelin-2 protein. **a** PDAC cells were collected for western blot analysis (left panel). The transgelin-2 protein level was normalized to actin level (right panel). *p < 0.05 compared with the BxPC-3 group. **b** SW1990, Capan-1 and Hs 766 T cells were co-transfected with *FASN* firefly luciferase reporter and renilla luciferase reporter for 24 h. The cells were then treated with 15uM U0126 (U0) for 6 h. The *FASN* firefly luciferase activity was normalized to the renilla luciferase activity. *p < 0.05 compared with the control (CT) group. **c-e**. SW1990, Capan-1 and Hs 766 T cells were treated with 25 uM fatostain (FA) for 3 h or 15uM U0126 (U0) for 6 h. Then RNA was purified and analyzed by qPCR. *p < 0.05 compared with the control (CT) group. **f-h**. SW1990, Capan-1 and Hs 766 T cells were treated with 25 uM fatostain (FA) for 3 h or 15uM U0126 (U0) for 6 h. The cells were then collected for western blot analysis (upper panel). The transgelin-2 protein level was normalized to actin level (lower panel). *p < 0.05 compared with the control (CT) group. **i**. SW1990, Capan-1, Hs 766 T and BxPc3 cells were treated with CHX for the indicated time points. The cells were then collected for western blot analysis. Transgelin-2 level was normalized by β-tubulin level. The normalized transgelin-2 level was expressed as a percentage of the initial level prior to the addition of CHX. *p < 0.05 compared with the BxPc3 group. **j**. SW1990 cells were treated with ERK1/2 inhibitor U0126 for 3 h. The cells were then treated with CHX for the indicated time points. The cells were then collected for western blot analysis. Transgelin-2 level was normalized by β-tubulin level. The normalized transgelin-2 level was expressed as a percentage of the initial level prior to the addition of CHX. *p < 0.05 compared with the control group

To elucidate the underlying mechanism of upregulation of transgelin-2, we examined the protein level of transgelin-2 in PDAC cells. As shown by ERK phosphorylation (Fig. 2a), the KRAS/ERK signaling pathway is activated in KRAS mutant cells. The protein level of transgelin-2 in KRAS mutant PDAC cells was significantly higher than that of wild type KRAS PDAC cells (Fig. 2a). We previously reported that sterol regulatory element binding protein (SREBP)-1 is responsible for the transcription of the *TAGLN2* gene [8]. Since KRAS mutation activate SREBP-1 transcriptional activity [20], KRAS is most likely to promote TAGLN2 gene transcription via SREBP-1. As expected, KRAS mutant cells showed higher SREBP-1 transcription activity than KRAS wild-type cells (Fig. 2b). Several studies have shown that SREBP-1 expression and activity are regulated by ERK [21–23]. SREBP-1 transcriptional activity was also significantly reduced in KRAS mutant cells, whereas ERK inhibition had minimal effect on SREBP-1 activity in KRAS wild-type cells (Fig. 2b). Therefore, the inhibition of SREBP-1 by fatostatin [24] significantly reduced the level of transgelin-2 mRNA in KRAS mutant PDAC cells (Fig. 2c-d), whereas the level of transgelin-2 protein was not completely blocked (Fig. 2f-g). However, the ERK inhibitor U0126 effectively reduced levels of transgelin-2 mRNA (Fig. 2c-d) and transgelin-2 protein (Fig. 2f-g) in KRAS mutant PDAC cells. In KRAS wild-type PDAC cells, SREBP-1 inhibition slightly decreased the protein level of transgelin-2 (Fig. 2h) due to transcriptional repression (Fig. 2e). In addition, ERK inhibition had no significant effect on the protein level of transgelin-2 in KRAS wild-type PDAC cells (Fig. 2h). The difference between protein and mRNA implied that KRAS might be involved in the transcriptional and post-transcriptional regulation of transgelin-2 expression. To further investigate whether KRAS is involved in the post-translational regulation of transgelin-2, we used cycloheximide (CHX) pulse-chase assay to analyze the stability of transgelin-2 protein. KRAS mutant cells showed significantly enhanced stability of the transgelin-2 protein as compared with KRAS wild-type cells (Fig. 2i). In addition, ERK inhibition significantly led to a decrease in the stability of the transgeline-2 protein (Fig. 2j). Taken together, these results support the role of KRAS-ERK signaling pathway in the transcriptional regulation of transgelin-2 protein.

### 29–31 amino acids of transgelin-2 are necessary for binding to ERK2

**Fig. 3:**
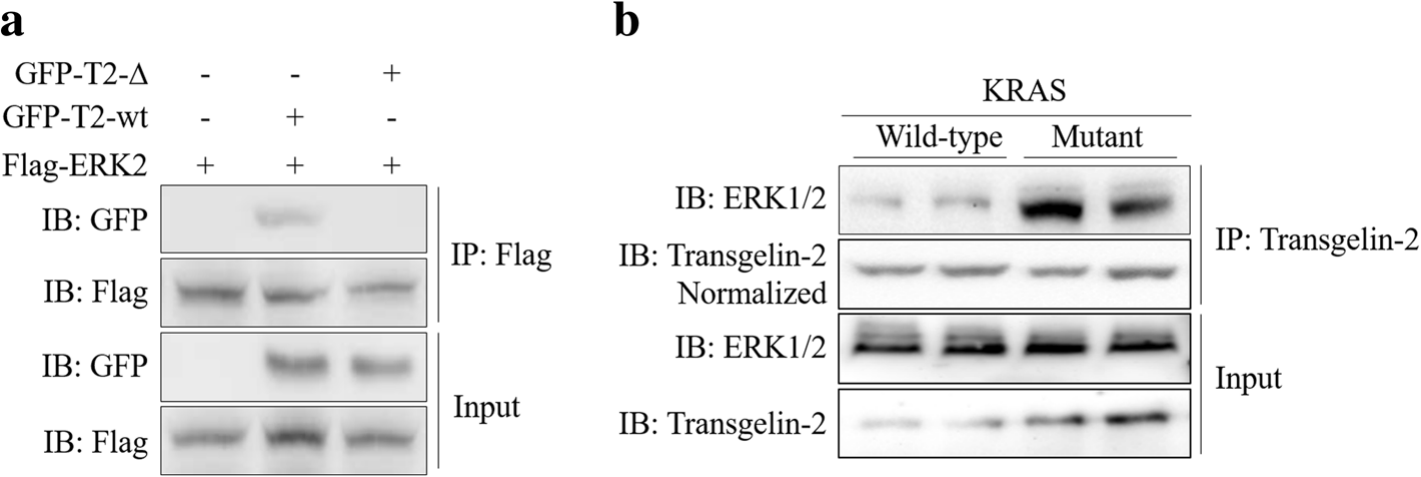
Transgelin-2 binds to ERK2 through its 29–31 aa. **a** HEK293T cells were co-transfected with either wild-type transgelin-2 (GFP-T2-wt) or 29–31 aa truncated mutant (GFP-T2-∆) and Flag-ERK2 for 48 h. Co-immunoprecipitation was performed by anti-Flag antibody. The immunoprecipitated proteins were analyzed by anti-GFP antibody to detect the presence of GFP-tagged transgelin-2. **b** KRAS wild-type cells (Hs 766 T and BxPc3) and mutant cells (SW1990 and Capan-1) were collected for co-immunoprecipitation assay. The cell lysates were immunoprecipitated by anti-transgelin-2 antibody. The immunoprecipitated proteins were analyzed by western blot using anti-ERK1/2 antibody

The core of KRAS signaling consists of a kinase cascade. Activated ERK then phosphorylates multiple downstream substrates [25, 26]. A key aspect of kinase-substrate recognition is the interaction of the kinase active site with the targeted phophosite [27]. In order to increase the selectivity of this interaction, mitogen-activated protein kinases (MAPKs) bind directly to short docking motifs on the substrate. The way of docking interactions is critical for MAPK cascade [28, 29]. The docking domain, also known as the LXL motif, has been identified as a binding motif that interacts with ERK [30]. Analysis of the transgelin-2 protein sequence identified the putative ERK binding sequence 24-ADLEQILIQWITT-36 containing the LXL motif at I29/I31. To assess whether transgelin-2 interacted with ERK2, wild-type or 29–31 truncated GFP-tagged transgelin-2 were co-transfected with Flag-tagged ERK2 into HEK293T cells. Co-immunoprecipitation analysis confirmed the interaction of transgelin-2 with ERK2 (Fig. 3a). The deletion of the ERK docking region (29-31aa) completely blocked the binding of transgelin-2 to ERK2, suggesting that the LXL motif is critical for their association (Fig. 3a). In addition, we found that protein-protein interaction between transgelin-2 and ERK were more pronounced in KRAS mutant cells than in KRAS wild-type cells (Fig. 3b). Taken together, these findings indicate that KRAS promotes the interaction between transgelin-2 and ERK.

### ERK phosphorylates transgelin-2 at serine-145 residue

**Fig. 4:**
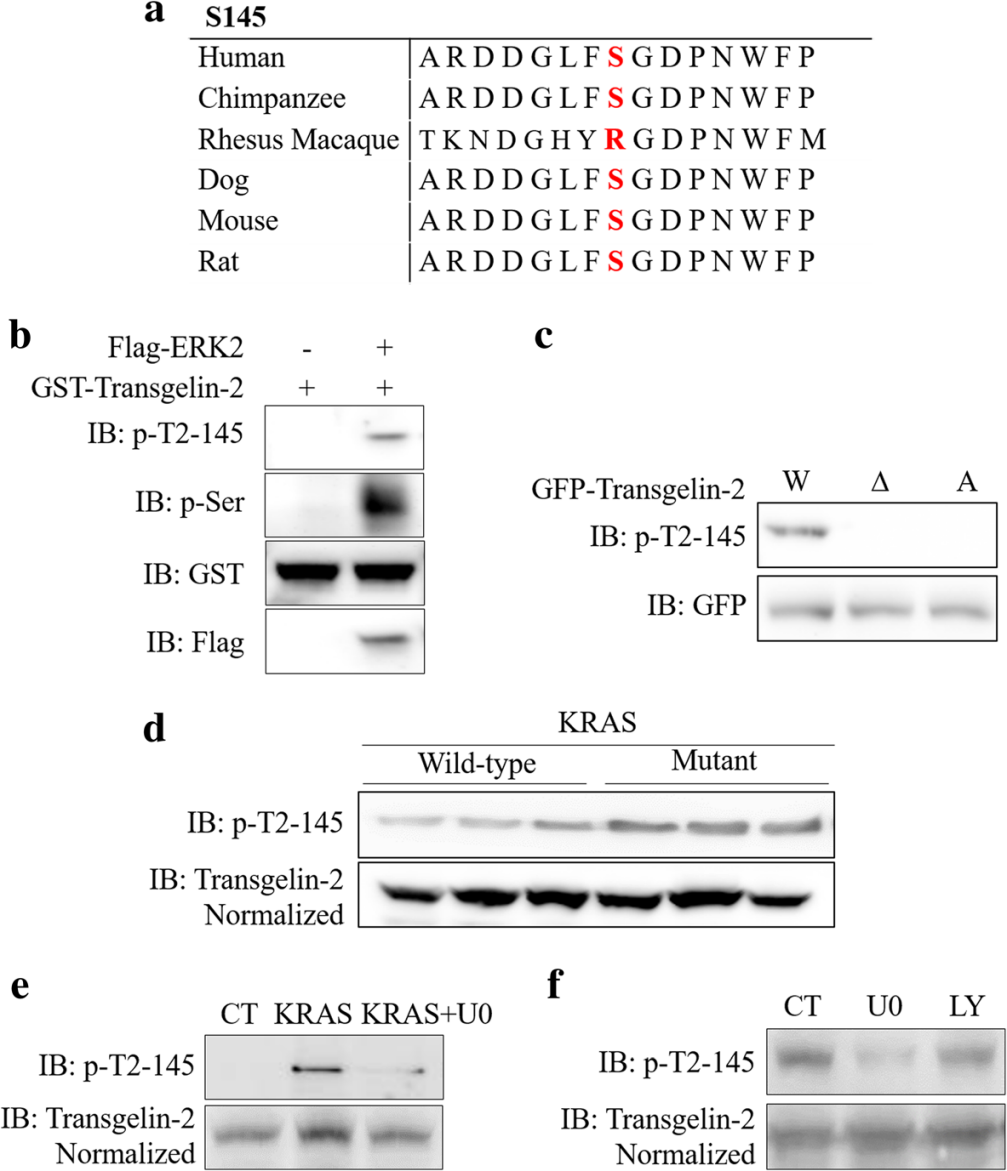
ERK2 phosphorylates transgelin-2 at S145 residue. **a**. The sequence of transgelin-2 in different species. **b**. In vitro kinase assay was performed using purified Flag-ERK2 and GST-transgelin-2. Phosphorylation of transgelin-2 at S145 was detected by anti-phospho-serine (p-ser) and anti-phospho-S145-transgelin-2 (p-T2-S145) antibodies. **c**. SW1990 cells were transfected with wild-type (W), 29-31aa truncated mutant (∆) or S145A mutant (A) of GFP-tagged transgelin-2 for 48 h. Phosphorylation of transgelin-2 on S145 was detected by anti-phospho-S145-transgelin-2 (p-T2-S145) antibody. **d**. KRAS wild-type cells (Hs 766 T, BxPc3 and Hs 700 T) and mutant cells (SW1990 Capan-1, and PANC-1) were collected for western blot assay. Phosphorylation of transgelin-2 on S145 was detected by anti-phospho-S145-transgelin-2 (p-T2-S145) antibody. **e**. HPDE6-C7 cells were transfected with KRAS^G12D^ mutant or vector control for 48 h. The cells were then treated with 15uM U0126 for 6 h. Phosphorylation of transgelin-2 on S145 was detected by anti-phospho-S145-transgelin-2 (p-T2-S145) antibody. **f**. SW1990 cells were treated with ERK1/2 inhibitor U0126 for 6 h or PI3K inhibitor LY294002 for 3 h. Phosphorylation of transgelin-2 on S145 was detected by anti-phospho-S145-transgelin-2 (p-T2-S145) antibody

As one of the RAS-MEK-ERK signaling cascade kinases, ERK regulates various proteins through phosphorylation [26]. Studies using stable isotope-based phosphoproteomics have revealed that serine-145 (S145) of transgelin-2 can be phosphorylated by ERK [31]. The S145 residue is also conserved among different species (Fig. 4a). To further confirm that transgelin-2 is a substrate for ERK, we performed in vitro kinase assay with purified Flag-ERK2 and GST-transgelin-2 proteins. The serine phosphorylation signal of transgelin-2 protein were clearly detected in the presence of ERK2 kinase (Fig. 4b). Using a specific antibody that recognized the phosphorylated S145, we observed that transgelin-2 was phosphorylated by ERK2 at the S145 residue (Fig. 4b). Mutation of serine to alanine (ie the S145A mutant) completely abolished the phosphorylation of the S145 residue (Fig. 4c). In addition, loss of ERK2 binding by truncation of 29–31 amino acids results in the inability of S145 residues to be phosphorylated (Fig. 4c). Since KRAS was able to regulate interaction between ERK and transgelin-2, phosphorylation of S145 in KRAS mutant cells was more pronounced than in wild-type cells (Fig. 4d). To test whether ERK-mediated phosphorylation of transgelin-2 was induced by KRAS, we expressed KRAS^G12D^ mutant in the untransformed normal human pancreas cell line HPDE6-C7. Expressing KRAS mutant significantly induced S145 phosphorylation of transgelin-2 (Fig. 4e). However, S145 phosphorylation was blocked by ERK inhibitor U0126 (Fig. 4e), suggesting that KRAS-induced transgelin-2 phosphorylation is mediated by ERK kinase. Since insulin and KRAS are known to activate the PI3K/AKT pathway, we analyzed the effect of PI3K inhibition on S145 phosphorylation. As shown in Fig. 4f, we observed that the PI3K inhibitor LY294002 had no effect on S145 phosphorylation, whereas the ERK inhibitor U0126 reduced the phosphorylation level of S145. Taken together, these data indicate that KRAS induces phosphorylation of transgelin-2 by ERK kinase.

### ERK stabilizes transgelin-2 protein by phosphorylating S145 residue

**Fig. 5:**
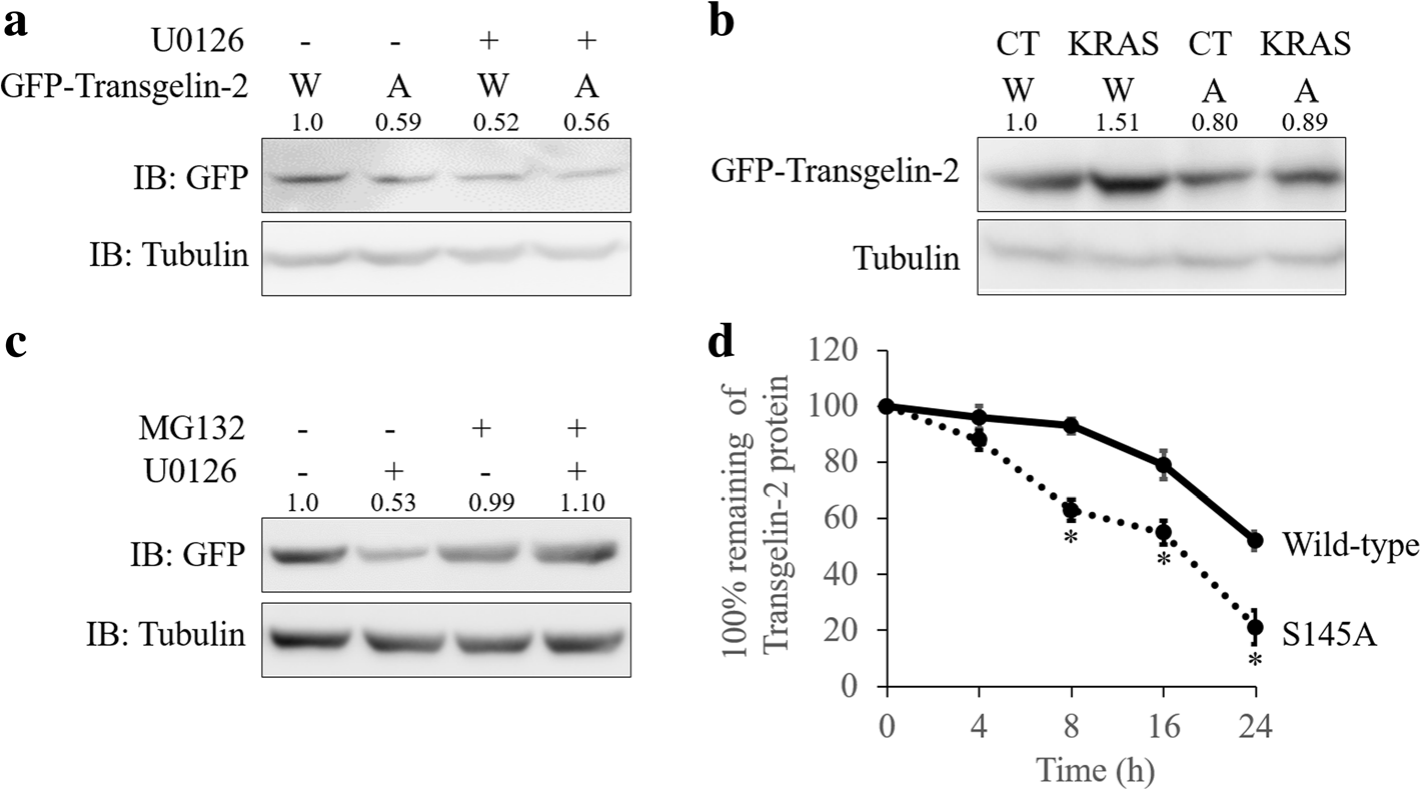
S145 phosphorylation stabilizes transgelin-2 protein. **a** SW1990 cells were transfected with wild-type (W) or S145A (A) mutant of GFP-tagged transgelin-2 for 48 h. The cells were then treated with U0126 for additional 6 h. Transgelin-2 protein level was assessed by western blot. **b**. KRAS^G12D^ (KRAS) or vector control (CT) were co-transfected with wild-type (W) or S145A (A) mutant of GFP-tagged transgelin-2 into HPDE6-C7 cells for 48 h. Transgelin-2 protein level was assessed by western blot. **c**. SW1990 cells were transfected with wild-type of GFP-tagged transgelin-2 for 48 h. The cells were then treated with 100 μM MG132 and/or 15uM U0126 for 6 h. Transgelin-2 protein level was assessed by western blot. **d**. SW1990 cells were transfected with wild-type or S145A mutant of GFP-tagged transgelin-2 for 24 h. The cells were then treated with CHX for the indicated time points. Transgelin-2 protein level was assessed by western blot. Transgelin-2 level was expressed as percentage of starting level prior to CHX addition. *p < 0.05 compared with the wild-type group

Since the protein turnover of the transgelin-2 was regulated by ERK, we tested whether S145 phosphorylation was involved in regulating the stability of the transgelin-2 protein. To test this, wild type or phosphorylation-deficient mutant (S145A) of transgelin-2 was expressed in PDAC cells. ERK inhibition reduced the protein level of wild-type transgelin-2, whereas phosphorylation-deficient mutant of transgelin-2 (S145A) was unaffected (Fig. 5a). In contrast, KRAS mutation significantly induced the accumulation of wild-type transgelin-2 protein, but did not induce accumulation of the S145A mutant (Fig. 5b). Next, we analyzed whether proteasome-mediated protein degradation was involved in the regulation of transgelin-2 protein. MG132, a proteasome inhibitor, rescued the reduction of transgelin-2 protein, suggesting that ERK regulates degradation of transgelin-2 (Fig. 5c). In addition, the S145 mutant has much lower protein stability compared with the wild-type (Fig. 5d). These data indicate that phosphorylation of transgelin-2 at the S145 residue regulates its protein stability.

### ERK-mediated phosphorylation of transgelin-2 regulates cell proliferation

**Fig. 6:**
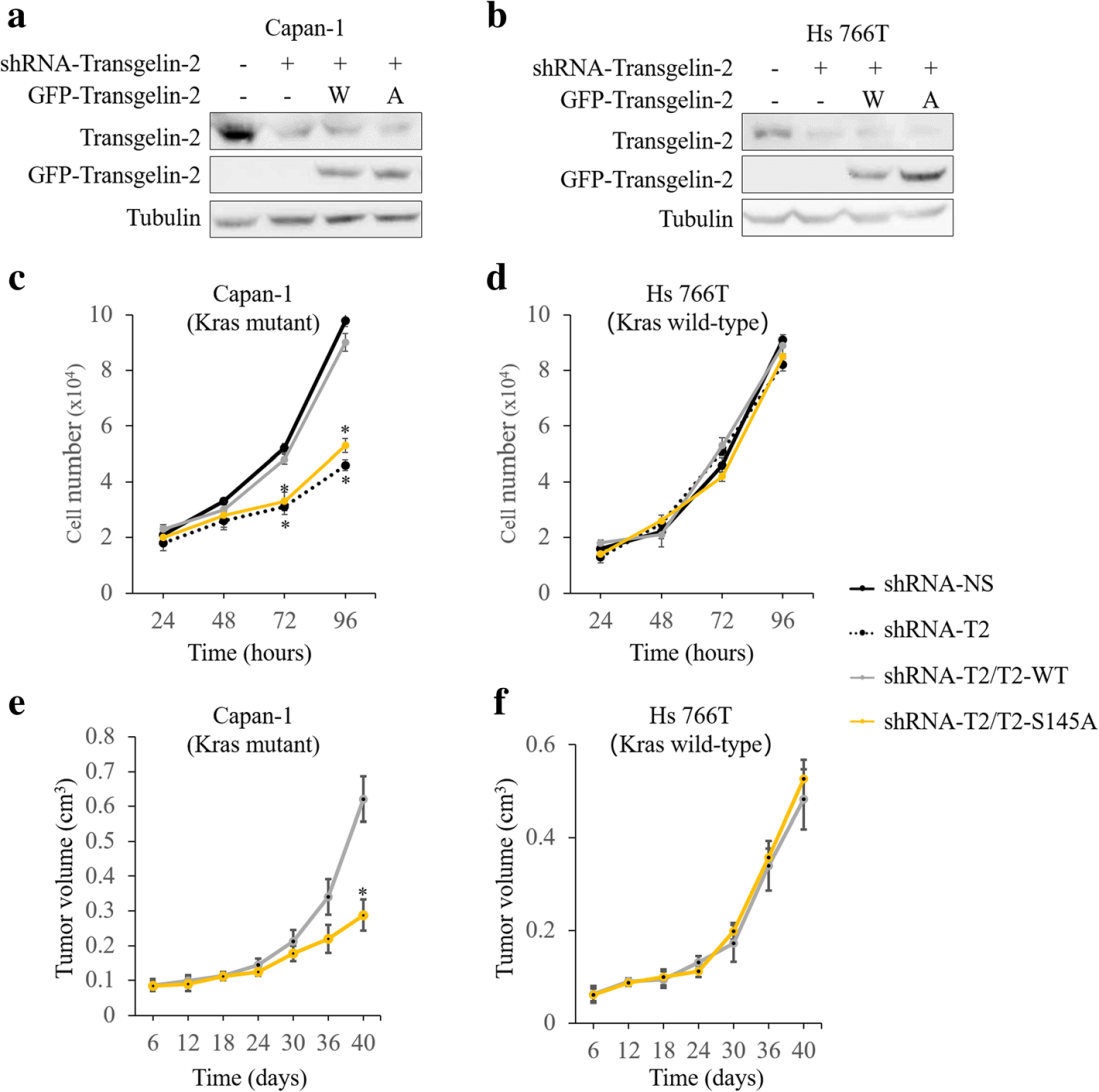
Transgelin-2 regulates cell proliferation in KRAS mutant PDAC cells. **a-b**. Transgelin-2 was stably silenced by shRNA against tranagelin2 (shRNA-T2) in Capan-1 and Hs 766 T cells. shRNA-NS was used as a control. The cells were then transfected with wild-type (W) or S145A mutant (A) of transgelin-2 for 16 h. The cells were then collected for western blot analysis. **c-d**. Transgelin-2 was stably silenced by shRNA against tranagelin2 (shRNA-T2) in Capan-1 and Hs 766 T cells. shRNA-NS was used as a control. The cells were then transfected with wild-type (T2-WT) or S145A mutant (T2-S145A) of transgelin-2 for 16 h. Growth curves were plotted based on the number of cells counted every 24 h. *p < 0.05 compared with the shRNA-NS group. **e-f**. Wild-type (shRNA-T2/T2-WT, *n* = 6) or S145 mutant (shRNA-T2/T2-S145A, *n* = 6) of transgelin-2 was overexpressed in transgelin-2 knockdown cells. The cells were then injected subcutaneously into nude mice. Tumor volume was measured at the indicated time points. *p < 0.05 compared with the shRNA-T2/T2-WT group

To analyze the role of S145 phosphorylation in cell proliferation, we ectopically expressed wild type or S145A mutant of transgelin-2 in PDAC cells (Fig. 6a-b). Knockdown of transgelin-2 significantly inhibits cell proliferation in KRAS mutant PDAC cells (Fig. 6c). Expression of wild-type of transgelin-2, but not S145 mutant, rescued proliferation defects in transgelin-2 knockdown cells (Fig. 6c). Moreover, knockdown of transgelin-2 had no significant effect on cell proliferation in KRAS wild-type cells (Fig. 6d). In KRAS wild-type cells, cell proliferation was comparable between wild type and S145A cells, suggesting that S145 phosphorylation of transgelin-2 is a downstream event of KRAS signaling (Fig. 6d). To evaluate the effect of S145 phosphorylation on cell proliferation in vivo, PDAC cells expressing wild-type or S145A mutant were injected subcutaneously into nude mice. In the KRAS mutation background, tumors derived from the S145 mutant cells were smaller than wild-type tumors (Fig. 6e), whereas tumors in both groups were comparable in size without KRAS mutation (Fig. 6f). These results indicate that the phosphorylation of transgelin-2 at S145 is essential for KRAS driven cell proliferation.

### S145 phosphorylation of transgelin-2 is a poor prognostic marker for PDAC

**Fig. 7:**
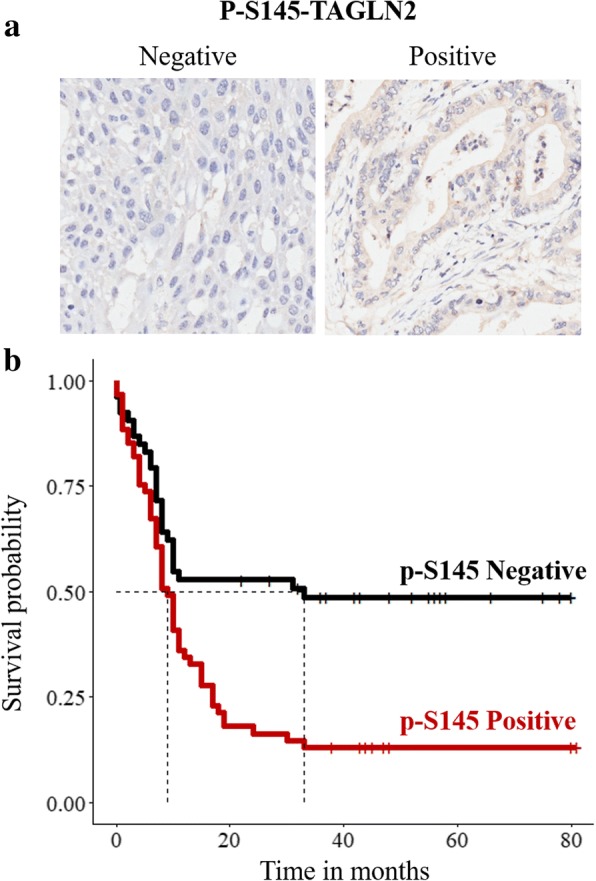
S145 phosphorylation of transgelin-2 predicts poor prognosis in patients with PDAC. **a**. Representative images of PDAC tissue stained with anti-phospho-S145-transgelin-2 (p-T2-S145) antibody. **b**. Survival analysis of PDAC patients according to level of anti-phospho-S145-transgelin-2. Dotted lines indicate the median survival time for each group

We examined phosphorylation level of S145 in the tumor tissues of 114 PDAC patients by immunohistochemistry. S145 phosphorylation of transgelin-2 was observed in the cytoplasm of PDAC cells (Fig. 7a). The correlation between S145 phosphorylation of transgelin-2 with clinicopathological characteristics was analyzed by chi-square test (Table 1). S145 phosphorylation of transgelin-2 was significantly associated with tumor stage (*p* = 0.027), tumor size (*p* = 0.02) and lymph node metastasis (*p* = 0.04), suggesting that transgelin-2 phosphorylation may be involved in the progression of PDAC. There was no significant correlation between S145 phosphorylation of transgelin-2 and other clinicopathological characteristics. Multivariate analysis showed that tumor size (*p* = 0.009) was still significantly associated with S145 phosphorylation of transgelin-2 (Table 2). Next, we examined whether there was correlation between phosphorylation level of S145 and patient survival. The survival rate of patients with positive staining of S145 phosphorylation was significantly lower than that of patients with negative staining (Fig. 7b). Of the 114 pancreatic cancer samples, 61 (54%) strongly expressed S145 phosphorylation of transgelin-2 and these patients had a median survival of only 9 months compared with 33 months in the 53 (46%) patients with weak staining (*p* = 0.004). Univariate survival analysis revealed that S145 phosphorylation of transgelin-2 (*p* = 0.001), T phase (*p* < 0.001), lymph node involvement (*p* = 0.002), and metastatic status (*p* = 0.003) were associated with survival in PDAC patients (Table 3). Multivariate Cox regression analysis showed that S145 phosphorylation of transgelin-2 (HR = 1.918, *p* = 0.008) and tumor stage (HR = 2.585, *p* = 0.004) were independent survival predictors (Table 4). Therefore, these results indicate that S145 phosphorylation of transgelin-2 is a potential prognostic indicator of PDAC.

**Table 1:** Univariate analysis of correlation between phosphorylation of transgelin-2 at S145 residue and clinicopathologocial factors

Variables	Total (n)	p-S145-transgelin-2 level		*p* value
		Positive n(%)	Negative n(%)	
Sex				
Male	69	39(64)	30(57)	0.544
Female	45	22(36)	23(43)	
Age at Surgery (years)				
< 65	66	38(62)	28(53)	0.406
≥ 65	48	23(38)	25(47)	
Tumour Differentiation				
Well/Moderate	79	38(62)	41(77)	0.125
Poor	35	23(38)	12(23)	
Tumor Stage				
I-II	53	22(36)	31(58)	0.027
III-IV	61	39(64)	22(42)	
Tumor location				
Head	87	48(79)	39(74)	0.676
Others	27	13(21)	14(26)	
Tumor Size				
< 2 cm	36	13(21)	23(43)	0.020
≥ 2 cm	78	48(79)	30(57)	
Metastasis status				
Patients with metastasis	2	2(3)	0(0)	0.539
Patients without metastasis	112	59(97)	53(100)	
Lymph Node Involvement				
Positve	58	37(61)	21(40)	0.040
Negative	56	24(39)	32(60)	
Vascular invastion				
Positve	72	40(66)	32(60)	0.705
Negative	42	21(34)	21(40)

**Table 2:** Multivariate analysis of correlation between phosphorylation of transgelin-2 at S145 residue and clinicopathologocial factors

Variable	Odds Ratio (95%) CI	*p* value
Tumor Stage (III-IV)	1.983 (0.671–5.994)	0.215
Lymph Node Involvement (yes)	1.601 (0.535–4.672)	0.393
Tumor Size (≥2 cm)	3.153 (1.363–7.636)	0.009

**Table 3:** Univariate analysis relationship between factors and survival

Variable	Hazard Ratio (95%) CI	*p* value
Sex	1.163 (0.734–1.843)	0.519
Age	1.091 (0.699–1.704)	0.702
Tumour Differentiation	1.398 (0.879–2.224)	0.157
Tumor Stage	2.805 (1.749–4.499)	< 0.001
Tumor location	1.102 (0.652–1.864)	0.716
Tumor Size	0.938 (0.587–1.499)	0.788
Metastasis status	9.482 (2.156–41.707)	0.003
Lymph Node Involvement	2.029 (1.292–3.188)	0.002
Vascular invastion	1.04 (0.659–1.64)	0.868
Transgelin2 p-S145	2.27 (1.42–3.627)	0.001

**Table 4:** Multivariate analysis relationship between factors and survival

Variable	Hazard Ratio (95%) CI	*p* value
Lymph Node Involvement (yes)	0.946 (0.508–1.761)	0.861
Tumor Stage (III-IV)	2.585 (1.345–4.968)	0.004
Transgelin-2 p-S145 (high)	1.918 (1.185–3.102)	0.008

## Discussion

Despite intensive research efforts to target KRAS, the impact of redundancy and compensation pathways limits the clinical use of these drugs in PDAC. KRAS mutations drive the oncogenesis of PDAC through a constitutively activated MAPK pathway. During PDAC development, different downstream effectors are activated or repressed by the MAPK pathway. Our results provide a novel mechanism of KRAS addiction in PDAC and link the ability of mutant KRAS to promote pancreatic cancer cell proliferation with transgelin-2.

Oncogenic KRAS signaling is the main driving force of PDAC development. KRAS signaling is highly complex and dynamic, involving a variety of downstream effectors. The oncogenic KRAS signaling in PDAC is believed to through the three major pathways: Raf/Mek/Erk, PI3K/Pdk1/AKT and Ral guanine nucleotide exchange factor (RalGEF) pathways [7]. Genetic inactivation of PDK1 is able to block KRAS-driven PDAC formation [32]. Activation of Raf/Mek/Erk by Braf^V600E^ with KRAS^G12D^ mutation results in a more aggressive phenotype with more PanINs compared with KRAS^G12D^ alone [33]. RalGEFs, which load GTP to small GTPases of the RAS superfamily, are necessary for KRAS-induced transformation of PDAC [34]. Here, our data indicate that KRAS downstream effector, ERK2, directly binds and phosphorylates transgelin-2. The protein stability of transgelin-2 is regulated by KRAS through ERK. It should mentioned that other KRAS downstream effectors may be also involved in regulation of transgelin-2.

Transgelin-2 is one of the homologues of transgelin, an early marker of smooth muscle cell (SMC) differentiation. Transgelin-2 in humans has 64% amino acid sequence homology to transgelin [35]. They have different cell-specific expression specificity. Transgelin is abundant in SMCs and fibroblasts, whereas transgelin-2 is predominantly expressed in epithelial cells. In PDAC tissues, both transgelin and transgelin-2 showed higher expression levels compared with adjacent normal tissues [8, 36, 37]. These studies establish a close relationship between the transgelin family and the development of PDAC. Due to the high similarity of protein sequences, it is worth further studying whether transgelin play similar biological roles as transgelin-2 in PDAC. Here, we found that the turnover of transgelin-2 protein is regulated through ERK-mediated phosphorylation upon KRAS mutation. The S145 residue in transgelin-2 is unique compared with its homolog, and so it is unlikely that transgelin is regulated by KRAS signaling in this way. Upregulation of transgelin in PDAC may be regulated by other upstream factors.

Several studies have shown that high levels of transgelin-2 in cancer tissues are due to down-regulation of specific microRNAs. The transgelin-2 gene is a target gene for miRNA-1 and miRNA-133a [38–41]. Most of these miRNAs are described as tumor suppressors and have the ability to inhibit cell proliferation by inhibiting transgelin-2. We have previously found that the transgelin-2 gene is a downstream gene for the SREBP-1 transcription factor. We observed that the inhibition of transgelin-2 gene transcription only partially inhibited the increase of transgelin-2 protein after KRAS activation. Therefore, the regulation of mRNA level does not completely explain the increase of transgelin-2 protein in PDAC cells. In fact, the transcription level in many cases is not sufficient to predict the protein level [42]. Here, we found that KRAS stabilized transgelin-2 protein by inhibiting proteasome-mediated degradation. Further evidence is needed to reinforce the existence of this posttranslational machinery in other types of cancer. Gene transcription, miRNA regulation, or protein stability regulation may have different effects on transgelin-2 in different types of cancers.

The function of transgelin-2 in cancer cells is largely unknown. However, in other types of cells, transgelin-2 can exert its function of stabilizing actin. Transgelin-2 is highly expressed in both T-cells and B-cells. In addition, transgelin-2 levels can be used to differentiate B cell subsets [43]. In T-cells, transgelin-2 stabilizes F-actin at the immunological synapse, thereby enhancing T cell activation and effector functions [44]. In B cells, transgelin-2 also participates in T cell activation by stabilizing T cell-B cell conjugation [45]. Transgelin-2 is also associated with nonalcoholic fatty liver disease (NAFLD), type 2 diabetes and hyperlipidemia. The levels of transgelin-2 are correlates with the severity of NAFLD [46, 47]. We have previously identified that transgelin-2 is highly expressed in PDAC patients with type 2 diabetes. Transgelin-2 is also a target of lipid master regulator SREBP-1 [8]. It appears that transgelin-2 is involved in the lipid metabolism of normal and malignant cells. Therefore, more insight should be provided on the molecular mechanism by which transgelin-2 regulates lipid metabolism. It is well-known that the homologue of transgelin-2, transgelin (SM22), plays an important role in migration and differentiation [48]. Several drugs including statins, Salvianolic acid A, Paeonol and SB-T-121205 regulate cancer cell metastasis and are associated with transgelin-2 expression [49–52]. Transgelin-2 is also involved in endothelial cell motility and tube formation, which involves the phosphorylation of myosin light chain followed by actin-myosin interaction [49]. In addition, transgelin-2 is up-regulated in stromal cells of lymph node-positive breast cancer [53]. Knockdown of transgelin-2 significantly inhibits invasion and metastasis of GBM cells. Mesenchymal related gene signatures are highly enriched in high transgelin-2 expression GBM tissues. And the mesenchymal phenotype of GBM cells can be reversed by transgelin-2 silencing [54]. This is consistent with our observation that lymph node involvement correlates with high levels of S145 phosphorylation of transgelin-2 in PDAC tissues. However, in multivariate analysis, there was no significant correlation between S145 phosphorylation of transgelin-2 levels and lymph node involvement. Tumor size remained significantly correlated with S145 phosphorylation of transgelin-2. Thus, transgelin-2 plays a dominant role in the regulation of cell proliferation in PDAC, which may be different in other types of cancer.

In summary, we found that transgelin-2 expression was regulated by KRAS in PDAC. KRAS mutation led to accumulation of transgelin-2 protein through phosphorylation of S145 residue by ERK. The S145 phosphorylation of transgelin-2 was a prognostic marker of PDAC. High level of S145 phosphorylation predicts poor prognosis in patients with PDAC. In addition, targeting KRAS-ERK-trasngelin2 can be utilized for PDAC treatment in the future.

## Conclusions

Transgelin-2 is a novel downstream target of KRAS-ERK signaling. Phosphorylation of transgelin-2 at S145 by ERK plays important roles in cell proliferation and tumorigenesis of PDAC. Increased S145 phosphorylation of transgelin-2 predicts poor prognosis of PDAC.

## Abbreviations

ERK: Extracellular signal regulated kinase
GBM: Glioblastoma multiforme
MAPK: Mitogen activated protein kinase
PDAC: Pancreatic ductal adenocarcinoma
RalGEF: Ral guanine nucleotide exchange factor
SMC: Smooth muscle cell
SREBP: Sterol regulatory element-binding protein
